# Breg Cells in Celiac Disease Isolated or Associated to Hashimoto's Thyroiditis

**DOI:** 10.1155/2018/5290865

**Published:** 2018-10-08

**Authors:** Maria Giulia Santaguida, Ilenia Gatto, Giorgio Mangino, Camilla Virili, Ilaria Stramazzo, Poupak Fallahi, Alessandro Antonelli, Patrizia Gargiulo, Giovanna Romeo, Marco Centanni

**Affiliations:** ^1^Department of Medico-Surgical Sciences and Biotechnologies, “Sapienza” University of Rome, Latina, Italy; ^2^Department of Clinical and Experimental Medicine, University of Pisa, Italy; ^3^Department of Experimental Medicine, “Sapienza” University of Rome, Italy; ^4^Endocrinology Unit, AUSL Latina, Latina, Italy

## Abstract

Hashimoto's thyroiditis (HT) may occur associated with celiac disease (CD). Regulatory B cells (Breg) subsets have been shown to play a significant role in autoimmune processes. Therefore, we have characterized their distribution in the peripheral blood obtained from 10 patients with isolated HT, 10 patients with HT + CD, 9 patients with isolated CD, and 9 healthy donors (HD). Th17 cells were significantly increased in patients with HT and in patients bearing both HT and CD, while patients with isolated CD exhibited a lower percentage of Th17, as compared with healthy donors. CD24^hi^CD38^hi^ Breg cells were significantly higher in patients with HT + CD and in patients with isolated CD as compared to both HD patients and patients with isolated HT (*p* = 0.0010). On the contrary, Breg memory phenotypes (CD24^hi^CD38^−^ and CD24^hi^CD27^+^) significantly decreased in patients with HT + CD as compared with the isolated disorders. Following CpG oligodeoxynucleotide stimulation, IL-10^+^ CD24^hi^CD38^hi^ Breg cells were similar in all groups of patients, despite these cells would have been higher in CD patients. In conclusion, celiac disease, isolated and even more when associated with HT, determines a peculiar behavior of Breg cells which are increased in number but possibly functionally defective. Furthermore, the association CD + HT was characterized by a reduction of Breg memory subsets as compared with the isolated disorders. The behavior of Th17 subset in patients with celiac disease associated with HT might have been sensitive to the effect of long-lasting GFD, and it is essentially determined by the presence of thyroid autoimmunity.

## 1. Introduction

Recent evidence has stressed that regulatory B (Breg) cells represent novel actors in maintaining the immune tolerance along with Th17 and regulatory T (Treg) cells [1, 2]. Breg cells, therefore, gained interest when studying autoimmune disorders [3]. The more frequent autoimmune disorder is autoimmune thyroid disease which may occur with other endocrine and nonendocrine autoimmune disorders [4–8]. One of the most relevant is represented by celiac disease (CD) which has been described in a significant number of patients with Hashimoto's thyroiditis (HT) [9, 10] as well as with other common autoimmune endocrine disorders [5, 10, 11]. The role of cellular immune response in the pathogenesis of these autoimmune disorders has been partially clarified [6–8]. As a matter of fact, in HT, a prevailing CD4^+^ Th1 polarization has been at first described [6, 7], but recently, it has been also described an involvement of Th17 cells in its pathogenesis [12, 13]. Th17 polarization is a characteristic of inflammatory phase of auto aggressive disorders and may precede Th1 phase of HT [12]. Cellular immune response is also relevant in CD pathogenesis: circulating Th17 cell levels were increased in adult patients, and they seemed to normalize upon appropriate gluten-free diet (GFD) [14]. Also, Treg cells, which maintain the peripheral tolerance [3], were increased in active CD and returned at normal levels after GFD [15]. Recently, it has been described that the CD19^+^ Breg cells may play a relevant role in dampening the T lymphocyte effector pathways Th1 and Th17 and enhancing Treg cells through the modulation of FoxP3 gene [16]. Breg cells' regulatory mechanism is far from being elucidated, despite the fact that production of IL-10 by some Breg subsets seems to be key in this network [17]. In fact, some different Breg subsets have been described, the main phenotypes being CD24^hi^CD38^hi^ Breg cells and memory Breg cells expressing CD27^+^on the cell surface [18, 19]. There are only few published studies on the characterization of Breg cells in patients with Hashimoto's thyroiditis either isolated [20, 21] or associated with other autoimmune disorders [22]. How the behavior of circulating Breg cells in celiac disease would be modified by the simultaneous presence of HT is not known and represents the aim of our study.

## 2. Patients and Methods

### 2.1. Patients

A total of 38 patients (34 women and 4 men; median age 45 years, IQ1-IQ3 = 35–45 years) were enrolled: 9 were healthy donors (HD), 10 patients were affected by isolated HT, 10 patients by CD and HT (CD + HT), and 9 by isolated CD. Their anthropometric and biochemical characteristics are shown in Table 1. The diagnoses have been established according to the specific guidelines or consensus [23, 24]. In particular, CD patients have been diagnosed before the age of 12. Celiac disease had been diagnosed by the presence of positive antiendomysial (EMA) and antitransglutaminase (tTG) antibodies and confirmed by endoscopy. Their histological damage has been classified as Marsh IIIa (*n* = 13) and Marsh IIIb (*n* = 6) [24]. They were slightly younger than the sample patients without celiac disease, and their levels of cholesterol and triglycerides were also slightly lower (Table 1). All CD patients were in gluten-free diet since almost 12 months, as demonstrated by the disappearance of clinical symptoms and the absence of previously positive antiendomysial (EMA) and antitransglutaminase (tTG) antibodies [25]. Patients with Hashimoto's thyroiditis had been diagnosed by the confirmed presence of antithyroperoxidase antibodies, the characteristic ultrasonographic pattern, and the presence of hypothyroidism [23]. All HT patients were subclinically hypothyroid and were similarly treated by titrating thyroxine dose individually, as previously described [9, 26]. At the time of sampling, HT patients were all euthyroid. In the whole sample, no patients had evidence of chronic disorders (diabetes, chronic obstructive pulmonary disease, obesity, renal failure, and cancer) nor infections and/or inflammatory disorders in the last 6 months and were neither pregnant nor nursing. Patients treated with drugs interfering with the immune response (steroids, immunosuppressant, and immune-modulatory and nonsteroidal anti-inflammatory drugs) were not enrolled. The study has been approved by the local Ethical Committee (Santa Maria Goretti Hospital, Latina, Italy) according to the local ethical rules and to the guidelines in the Declaration of Helsinki. Written informed consent from all patients was obtained before the beginning of the study.

### 2.2. Methods

PBMCs were collected from all enrolled subjects as already reported [22]. Fresh PBMCs were stained with specific antibodies. The characterized phenotype of Th17 was CD4^+^ IL17A^+^, and the results are expressed as percentages of total CD4^+^ T lymphocytes.

The results for B cell subset analysis are expressed as percentages of total CD19^+^ B lymphocytes. The following phenotypes were characterized:

- Total B cells = CD19^+^
- Breg cells = CD19^+^CD24^hi^CD38^hi^
- “Primary” Breg memory cells = CD19^+^CD24^hi^CD38^neg^
- Breg memory cells = CD19^+^CD24^hi^CD27^+^

For both T and B cells, cells were stimulated with PMA, Ionomycin, Brefeldin A, and Monensin (PIB) (cell stimulation cocktail plus protein transport inhibitor, Affymetrix eBioscience, San Diego, CA, USA) in the last 5 hours.

T cells were surface-stained with anti-CD4 FITC antibody, fixed, permeabilized, and stained intracellularly with anti-IL-17A PE antibody (Affymetrix eBioscience, San Diego, CA, USA).

B cells were surface-stained with anti-CD19 FITC, anti-CD24 APC-eFluor 780, anti-CD38 APC, and anti-CD27 PerCPcy5.5 antibodies, fixed, permeabilized, and intracellular-stained with anti-IL-10 PE antibody (Affymetrix eBioscience, San Diego, CA, USA).

To evaluate IL-10 production by Breg cells, PBMCs were incubated with 0.1 *μ*M CpG-B ODN2006 (InvivoGen, San Diego, CA, USA) for 72 hours at 37°C.

CpG motifs contain a cytosine (C) followed by a guanine (G) triphosphate deoxynucleotide with a phosphodiester (p) link. CpG oligodeoxynucleotides are short single-stranded synthetic DNA immunostimulants that have been considered pathogen-associated molecular pattern (PAMP) molecules. They are recognized by the pattern recognition receptor (PRR) Toll-Like Receptor 9 (TLR9) [22]. As a negative control, PBMCs were also incubated with non-CpG ODN 2006 Control (ODN 2137, InvivoGen, San Diego, CA, USA) in the same conditions. Moreover, the intracellular staining was performed with an appropriate isotype control Abs for gate setting (Affymetrix eBioscience, San Diego, CA, USA).

Data from these phenotypes are expressed as percentages of IL-10-positive cells referred to the corresponding B lymphocyte subsets.

- Total B cells IL10^+^ = CD19^+^ IL10^+^
- IL10^+^ Breg cells = CD19^+^CD24^hi^CD38^hi^ IL10^+^
- “Primary” IL10^+^ Breg memory cells = CD19^+^CD24^hi^CD38^neg^ IL10^+^
- IL10^+^ Breg memory cells = CD19^+^CD24^hi^CD27^+^ IL10^+^

Cells were acquired on a FACS ARIA II flow cytometer (Becton Dickinson, San Jose, CA, USA). At least 10,000 events were acquired on CD4^+^ or CD19^+^ gate. FACS Diva software (v6.11, Becton Dickinson) was used for analysis.

### 2.3. Statistical Analysis

Results are expressed as median value. The difference among more than two groups was calculated using nonparametric Kruskal-Wallis test and Dunn posttest to compare all pairs of data.

INSTAT GraphPad Prism 5.0 software for Windows (GraphPad, La Jolla, Ca. USA) was used for the statistical analysis.

## 3. Results

### 3.1. Th17 Lymphocytes

**Figure 1:**
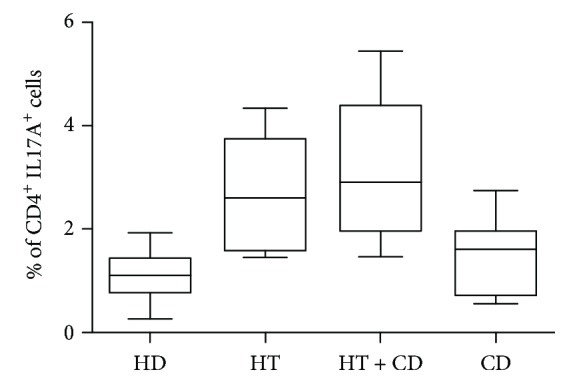
Th17. Percentage of Th17 cells (CD4 + IL17A+) in healthy donors (HD), in patients with Hashimoto thyroiditis (HT), in patients with Hashimoto thyroiditis plus celiac disease (HT + CD), and in patients with celiac disease (CD). Box plots indicate median, interquartile range (box), and minimum and maximum values (whiskers). Nonparametric Kruskal-Wallis test *p* = 0.0011. Dunn posttest: HD vs HT  *p* < 0.05; HD vs HT + CD *p* < 0.01; HT + CD vs CD *p* < 0.05.

The median percentage of Th17 cells was different in each group of patients (Kruskal-Wallis test: *p* = 0.0011). As expected, Th17 cells were significantly increased in patients with isolated HT, as compared with healthy donors. In patients bearing both HT and CD, the difference with healthy donors was even greater, while patients with isolated CD exhibited a lower percentage of Th17 (Figure 1).

### 3.2. B Lymphocyte Subsets

Total CD19^+^ B lymphocytes were similar in all groups of patients (*p* = 0.3032, data not shown).

#### 3.2.1. CD24^hi^CD38^hi^ Bregs

**Figure 2:**
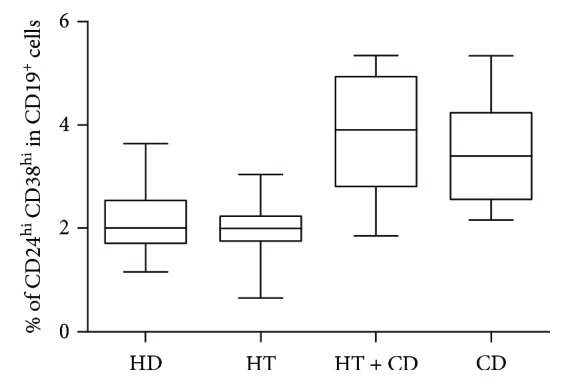
Nonstimulated Breg cells. Percentage of Breg cells (CD24^hi^ CD38^hi^) in healthy donors (HD), in patients with isolated Hashimoto thyroiditis (HT), in patients with Hashimoto thyroiditis plus celiac disease (HT + CD), and in patients with celiac disease (CD). Box plots indicate median, interquartile range (box), and minimum and maximum values (whiskers). Nonparametric Kruskal-Wallis test *p* = 0.0010. Dunn posttest: HD vs HT + CD *p* < 0.05; HT vs HT + CD *p* < 0.01; HT vs CD *p* < 0.05.

The analysis of this subset revealed significant differences (Kruskal-Wallis test: *p* = 0.001) (Figure 2). The median percentage of CD24^hi^CD38^hi^ Breg cells was 2.0% both in HD patients and in patients with isolated HT. Higher percentages were instead recorded in patients with isolated CD and in those with CD + HT which were, however, similar each other (*p* = ns). In particular, patients bearing both HT and CD showed significantly higher Breg subset than HD and HT patients (Figure 2).

#### 3.2.2. Memory Breg Subsets

**Figure 3:**
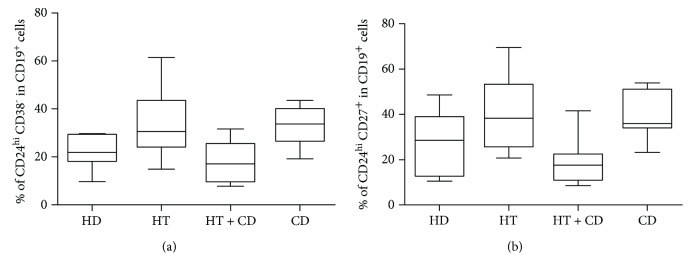
Nonstimulated Breg memory cells. (a) Percentage of Breg memory cells (CD24^hi^ CD38^−^) in healthy donors (HD), in patients with isolated Hashimoto thyroiditis (HT), in patients with Hashimoto thyroiditis plus celiac disease (HT + CD), and in patients with celiac disease (CD). Box plots indicate median, interquartile range (box), and minimum and maximum values (whiskers). Nonparametric Kruskal-Wallis test *p* = 0.0065. Dunn posttest: HT vs HT + CD *p* < 0.05 and HT + CD vs CD *p* < 0.05. (b) Percentage of Breg memory cells (CD24^hi^ CD27^+^) in healthy donors (HD), in patients with isolated Hashimoto thyroiditis (HT), in patients with Hashimoto thyroiditis plus celiac disease (HT + CD), and in patients with celiac disease (CD). Box plots indicate median, interquartile range (box), and minimum and maximum values (whiskers). Nonparametric Kruskal-Wallis test *p* = 0.0122. Dunn posttest: HT vs HT + CD *p* < 0.05 and HT + CD vs CD *p* < 0.05.

The median percentage of the “primary” memory Breg cells (CD19^+^CD24^hi^CD38^neg^cells) was significantly decreased in CD + HT patients, being statistically different from isolated HT and CD (Kruskal-Wallis test: *p* = 0.0065) (Figure 3(a)).

These findings have been mirrored by the ones obtained when CD19^+^CD24^hi^CD27^+^ memory cells were measured. Again, the percentage of these memory cells was lower in patients with CD + HT than in patients bearing the two diseases in isolated form (Kruskal-Wallis test: *p* = 0.0122) (Figure 3(b)). This may imply that memory CD24^hi^CD38^neg^ and CD27^+^ B cells overlapped; in fact, we found that more than 2/3 of CD19^+^CD27^+^ cells also include the phenotype CD24^hi^CD38^neg^.

### 3.3. CpG Stimulation

#### 3.3.1. CD24^hi^CD38^hi^ Bregs

**Figure 4:**
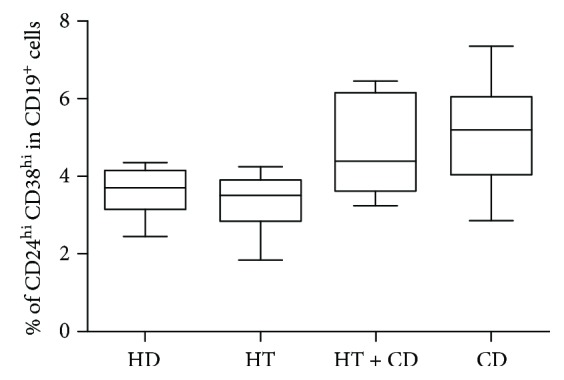
Stimulated IL-10^+^ Breg cells. Percentage of IL10^+^ Breg cells (CD24^hi^ CD38^hi^ IL10^+^) after CpG stimulation in healthy donors (HD), in patients with isolated Hashimoto thyroiditis (HT), in patients with Hashimoto thyroiditis plus celiac disease (HT + CD), and in patients with celiac disease (CD). Box plots indicate median, interquartile range (box), and minimum and maximum values (whiskers). Nonparametric Kruskal-Wallis test *p* = 0.0159. Dunn posttest: HT vs CD *p* < 0.05. Analysis of IL-10-producing cells was performed on the previously shown B lymphocyte subsets (CD24^hi^ CD38^hi^ cells).

Following CpG stimulation, total CD19^+^cells were different among groups (Kruskal-Wallis *p* = 0.0098), and in particular, patients with isolated CD showed a significant reduction of this phenotype as compared to the one observed in HD patients and in patients with HT + CD (1.6% vs 3.9% or 4.2%; both *p* < 0.05). Even the proportions of CD24^hi^CD38^hi^ Bregs were significantly different among groups (*p* = 0.0159), the Breg fraction being higher in patients with isolated CD (Figure 4). On the contrary, when these CpG-stimulated lymphocytes were stratified by their IL-10 production, no differences were observed among groups (*p* = ns, data not shown).

#### 3.3.2. Memory Breg Subsets

Small but significant differences were observed in memory CD19^+^CD24^hi^CD38^neg^ cells following CpG stimulation (*p* = 0.0362). A decreased percentage of this subset was observed in CD + HT patients as compared to the one observed in isolated HT (*p* < 0.05). The behavior of this memory subset seems to mirror the one in nonstimulated Breg. On the contrary, the percentage of CD19^+^CD27^+^ cells was similar in all subgroups (*p* = ns). Furthermore, IL-10 expression after CpG stimulation was similar in both subgroups of memory Breg cells (data not shown).

## 4. Discussion and Conclusion

Celiac disease and Hashimoto's thyroiditis often occur associated [4, 5, 9, 10] and share some immunologic similarities [9]. It appears to be useful, therefore, to look for immunologic variations when the two disorders coexist in the same patients.

These data show that, in patients with CD, the percentage of TH17 cells was low and similar to the one observed in healthy donors, as expected in gluten-free diet patients in whom the inflammatory damage is no longer active [14]. In contrast, there was a significant increase of TH17 cells in patients with HT [12] even when it coexists with CD. The percentage of nonstimulated CD24^hi^CD38^hi^ Breg cells was increased in patients with CD even when associated with HT but not in patients with isolated HT, thus indicating that CD may enhance this regulatory response, as described for chronic intestinal inflammatory conditions by Mizoguchi et al. [27]. We also observed a small but significant decrease percentage of both primary CD24^hi^CD38^neg^ Breg cells and memory Breg cells (CD24^hi^CD27^+^) in patients with CD + HT. In fact, when CD and HT concurred, memory Breg cells were significantly lower than in patients with both isolated disorders, indicating that the association of these autoimmune disorders exerted an opposite effect on Breg and Breg memory cells. This is in keeping with our previous results [22], since we described that Breg cells are quantitatively increased in patients with HT and further autoimmune disorders; however, in this study, we have shown that this effect was seen even in patients with isolated CD, showing a prevailing role of this disease over HT on the expression of CD24^hi^CD38^hi^ Breg cells. This was not the case for Breg memory cells, where the copresence of HT and CD resulted in a negative synergistic effect, leading to a clearly reduced CD19^+^CD24^hi^CD38^neg^ subset. Again, these results are in keeping with our previous study with multiple auto aggressive disorders associated with HT [22].

It has been reported that naïve and memory B cells are distinguished by the production of different pro- and anti-inflammatory cytokines: naïve B cells mainly produce anti-inflammatory cytokines, whereas memory B cells are the main responsible for proinflammatory cytokine production [28–30]. IL-10 production has been considered as a proof of functionality of Breg cells [1, 2], being enhanced *in vitro* by CpG and/or other stimuli. In a previous study from Kristensen et al. [20], CpG stimulus increased Breg cell subsets in HT patients, but the effect was similar to that observed in healthy donors. On the contrary, in our previous study, CpG stimulus increased IL10^+^ Bregs in HT patients significantly more than in healthy donor, but no further differences were observed in patients with multiple autoimmune disorders [22]. Similar results were obtained in our present study in patients with HT and celiac disease that did not show any change in the percentage of IL10^+^ Bregs despite the increased number of these cells. Noticeably, a recent paper from Yu et al. [21] described the lack of CD24^hi^CD38^hi^ IL10^+^cells associated with a reduced regulatory activity and inability to suppress inflammatory cytokines in patients with HT. However, this finding, even in our patients, cannot rule out the regulatory effects of Bregs through other pathways (i.e., TGFbeta) as previously described [28]. A further issue to be kept on mind about our study group, as well as in general, is the phase of autoimmune disorders in which the patients have been studied. In fact, our patients were all in GFD since at least 12 months which usually reduces the inflammatory phase at the level of duodenal mucosa [14, 17]. Both CD and HT are considered autoimmune disorders with an initial Th17 polarization [12, 31, 32] which activity may be counteracted by increased IL10^+^Breg cells, leading to proinflammatory cytokine suppression [33]. Our patients were clearly outside the inflammatory phase of CD, and the patients with HT were euthyroid because they are treated with levothyroxine, whose anti-inflammatory effect has been recently described [34]. Therefore, our patients are supposed to be in a phase in which inflammation is not the prevailing characteristic. The reason why the memory subsets are furthermore reduced in patients with HT + CD, as compared to what is found in the isolated forms of diseases, deserves further studies.

In conclusion, in patients with celiac disease, isolated and even associated with HT, there emerges a peculiar behavior of Breg cells which are increased in number but possibly functionally defective. Furthermore, the association CD + HT was significantly associated with a reduction of Breg memory subsets as compared with the isolated disorders. Finally, the behavior of Th17 subset in patients with celiac disease associated with HT might have been sensitive to the effect of long-lasting GFD, and it is essentially determined by the presence of thyroid autoimmunity.

## Figures and Tables

**Table 1:** Anthropometric and biochemical characteristics of patients.

	HD	HT	HT + CD	CD	*p*
Subjects (no.)	9	10	10	9	N/A
Sex (women/male)	8W/1M	8W/2M	9W/1M	9W	N/A
Age (years) median (IQ1-IQ3)	49 (39–54)	47 (42–57)	39 (22–49)	37 (29–44)	ns
Weight (Kg) median (IQ1-IQ3)	67 (60–72)	67 (60–79)	54 (51–58)	60 (56–72)	0.0448
Total cholesterol (mg/dL) median (IQ1-IQ3)	190 (173–210)	201 (186–226)	177 (147–201)	174 (150–198)	ns
Triglycerides (mg/dL) median (IQ1-IQ3)	101 (90–126)	120 (100–136)	82 (61–110)	77 (57–109)	0.0431
TSH at first examination (mU/L) median (IQ1-IQ3)	1.20 (0.90–2.1)	12.80 (10.96–17.7)	14.50 (12.95–16.52)	1.48 (0.99–2.13)	N/A
TSH under L-T4 therapy (mU/L) median (IQ1-IQ3)	N/A	1.22 (0.96–1.72)	1.10 (0.95–1.52)	N/A	ns

HT = Hashimoto's thyroiditis; CD = celiac disease.

## Data Availability

We retain the original data and FACS data and graphs which are available to any request.
